# All About (NK Cell-Mediated) Death in Two Acts and an Unexpected Encore: Initiation, Execution and Activation of Adaptive Immunity

**DOI:** 10.3389/fimmu.2022.896228

**Published:** 2022-05-16

**Authors:** Ariel Ramírez-Labrada, Cecilia Pesini, Llipsy Santiago, Sandra Hidalgo, Adanays Calvo-Pérez, Carmen Oñate, Alejandro Andrés-Tovar, Marcela Garzón-Tituaña, Iratxe Uranga-Murillo, Maykel A. Arias, Eva M. Galvez, Julián Pardo

**Affiliations:** ^1^ Immunotherapy, Inflammation and Cancer, Aragón Health Research Institute (IIS Aragón), Biomedical Research Centre of Aragón (CIBA), Zaragoza, Spain; ^2^ Unidad de Nanotoxicología e Inmunotoxicología (UNATI), Centro de Investigación Biomédica de Aragón (CIBA), Aragón Health Research Institute (IIS Aragón), Zaragoza, Spain; ^3^ Centro de Investigación Biomédica en Red en Enfermedades Infecciosas (CIBERINFEC), Instituto de Salud Carlos III (ISCIII), Zaragoza, Spain; ^4^ Instituto de Carboquimica (ICB), CSIC, Zaragoza, Spain; ^5^ Department of Microbiology, Preventive Medicine and Public Health, Fundación Agencia Aragonesa para la Investigación y el Desarrollo ARAID Foundation, University of Zaragoza, Zaragoza, Spain

**Keywords:** NK cells, cell death, granzymes, perforin, granule exocytosis, death receptors, immunological cell death

## Abstract

NK cells are key mediators of immune cell-mediated cytotoxicity toward infected and transformed cells, being one of the main executors of cell death in the immune system. NK cells recognize target cells through an array of inhibitory and activating receptors for endogenous or exogenous pathogen-derived ligands, which together with adhesion molecules form a structure known as immunological synapse that regulates NK cell effector functions. The main and best characterized mechanisms involved in NK cell-mediated cytotoxicity are the granule exocytosis pathway (perforin/granzymes) and the expression of death ligands. These pathways are recognized as activators of different cell death programmes on the target cells leading to their destruction. However, most studies analyzing these pathways have used pure recombinant or native proteins instead of intact NK cells and, thus, extrapolation of the results to NK cell-mediated cell death might be difficult. Specially, since the activation of granule exocytosis and/or death ligands during NK cell-mediated elimination of target cells might be influenced by the stimulus received from target cells and other microenvironment components, which might affect the cell death pathways activated on target cells. Here we will review and discuss the available experimental evidence on how NK cells kill target cells, with a special focus on the different cell death modalities that have been found to be activated during NK cell-mediated cytotoxicity; including apoptosis and more inflammatory pathways like necroptosis and pyroptosis. In light of this new evidence, we will develop the new concept of cell death induced by NK cells as a new regulatory mechanism linking innate immune response with the activation of tumour adaptive T cell responses, which might be the initiating stimulus that trigger the cancer-immunity cycle. The use of the different cell death pathways and the modulation of the tumour cell molecular machinery regulating them might affect not only tumour cell elimination by NK cells but, in addition, the generation of T cell responses against the tumour that would contribute to efficient tumour elimination and generate cancer immune memory preventing potential recurrences.

## Introduction

Natural killer (NK) cells are effector cells of the innate immune system that play a key role in the control of intracellular pathogens and tumours, especially against those that evade adaptive immunity by interfering with MHC-mediated antigen presentation (1). Indeed, unlike its counterparts in the adaptive immune system, cytotoxic CD8 T cells, NK cells preferentially eliminate cells that have downregulated MHC expression. NK cells sense their environment through both cytokine receptors and germline-encoded activating and inhibitory receptors specific for endogenous danger or pathogen signals. Cytokine receptors are mainly involved in NK cell proliferation and acquisition of functional molecules (including receptors and cytotoxic and immunoregulatory molecules) and trafficking to inflamed tissues where they will search for affected cells; while activating and inhibitory receptors modulate NK cell activation and recognition and elimination of target cells, which will be the main focus of this review.

NK cells are professional killer cells that recognize and rapidly destroy cells that are dangerous to the host, like stressed, infected, or transformed cells, contributing to viral and cancer immune surveillance (2, 3). NK cells are a heterogeneous and plastic population acquiring different phenotypes and functions depending on the tissue context and signalling cues they are exposed to. In general, human NK cells are phenotypically identified by the expression of CD56 in the absence of CD3 (4, 5).

Human NK cells are mainly classified into CD56^Bright^ and CD56^Dim^ subsets based on cell-surface CD56 density and their cytotoxic potential; these subsets differ in function, phenotype, and tissue localization. CD56^Dim^ subset represents more than 90% of peripheral blood NK (pNK) cells and expresses high levels of the cytotoxic molecules perforin and granzyme B as well as the activating CD16 IgG Fc receptor and different members of killer Ig-like receptors (KIRs). They are highly cytotoxic and also produce cytokines after recognizing activating ligands expressed on target cells. In contrast, the subset of CD56^Bright^ NK cells is rare in blood and preferentially reside in secondary lymphoid organs, such as lymph nodes, and express low perforin, granzyme B and killer Ig-like levels receptor, responding with strong cytokine and chemokine production to soluble factors (6, 7).

**Figure 1:**
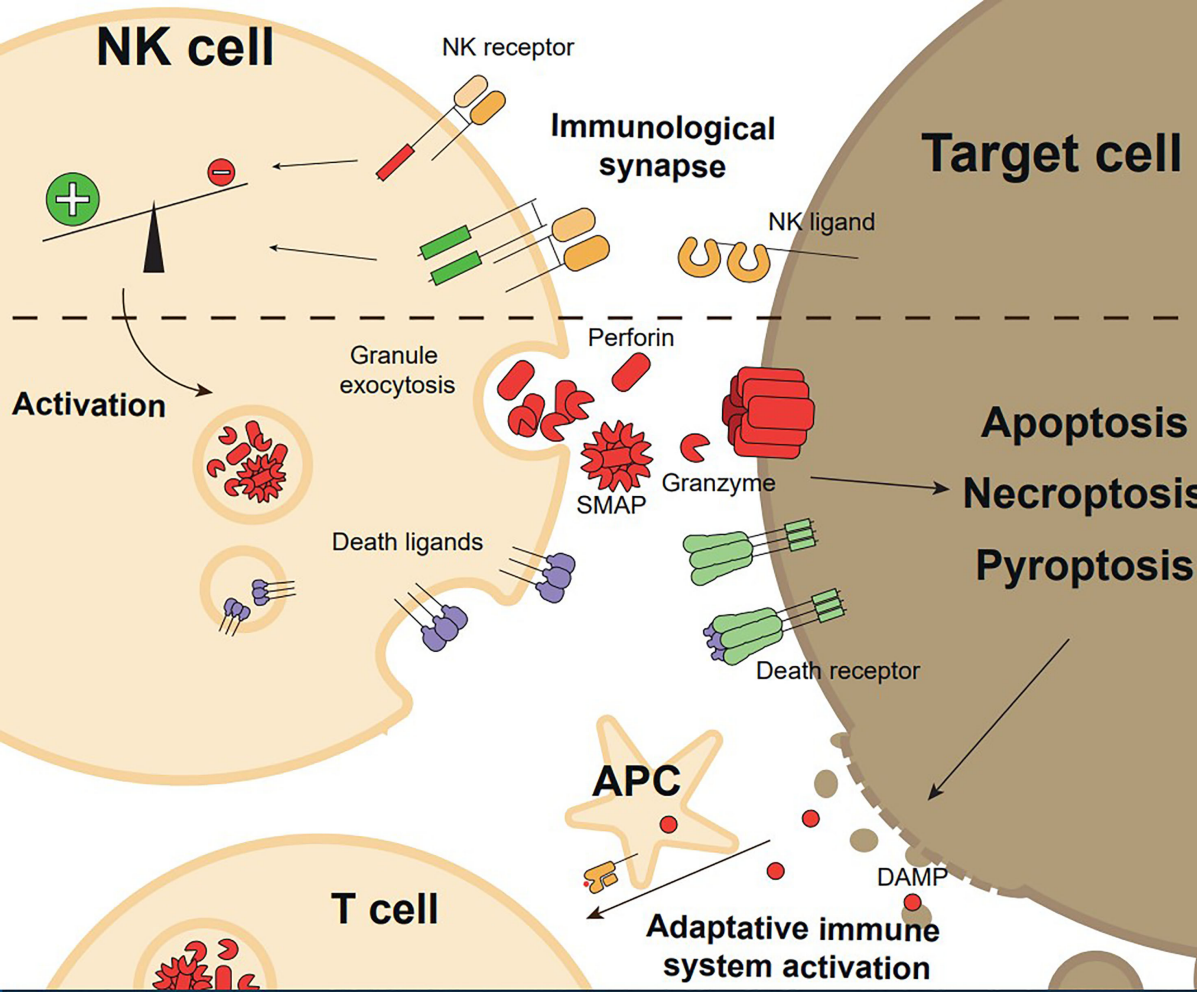
Overview of the execution and functional consequences of NK cell mediated cytotoxicity. Target cell recognition through NK cell receptor-ligand interaction and formation of the immunological synapse with a pro-activating signals balance promote NK cell activation. Upon activation, NK cells exert their effector functions, granule exocytosis, or expression of death ligands, inducing target cell killing by regulated cell death (apoptosis, necroptosis, or pyroptosis). Target cell death generates Danger Associated Molecular Patterns (DAMPs) and releases tumour antigens, which induces adaptative immune system activation.

Despite this heterogeneity, NK cells are still mainly recognized by their ability to kill infected and transformed cells, a process known as NK cell-mediated cytotoxicity. Activation of NK cell-mediated cytotoxicity is controlled by the balance between inhibitory and activating signals transduced by several inhibitory and activating receptors specific for pathogen and/or host cell derived ligands (1–3). Once NK cells are activated and recognize the target cell, they will make use of an arsenal of molecular mechanisms capable of executing target cell death. The main mechanisms involved in NK cell-mediated cytotoxicity are the granule exocytosis pathway (perforin/granzyme) and the expression of death ligands (8), which have been traditionally described to activate apoptotic target cell death. However, more recent, indirect evidence suggests that other types of programmed cell death could be induced by NK cells like necroptosis or pyroptosis, leading to enhanced inflammatory responses (9). The final consequence of this process is not only the elimination of the target cell, but in addition, depending on the way how target cells die, it will modulate secondary adaptive immune responses that will further enhance pathogen or tumour elimination (9). This review will present and discuss the key mechanisms used for NK cells to regulate and induce cell death in three parts (**Figure 1**): 1- signals required to recognize the target cell and activate the NK cell intracellular cytotoxic machinery; 2- the role of different cell death pathways activated in the target cells during NK-cell mediated killing and 3- the emerging evidence on how cell death induced by NK cells might be a novel immunoregulatory mechanism linking innate and adaptive immune responses.

For reasons of clarity and length, in this review, we will focus on the main mechanisms involved in the cytotoxic function mediated by NK cells and their biological relevance, without analyzing the function of these mechanisms in other cells that present similar effector molecules such as CD8^+^Tc cells. Other excellent reviews have presented a more general view of some of these mechanisms, not only focused on NK cells (8, 10–13).

## First Act: Judges and hangmen in NK Cell-Mediated Cytotoxicity

### Specific Receptors Dictate the Killing of Target Cells

**Figure 2:**
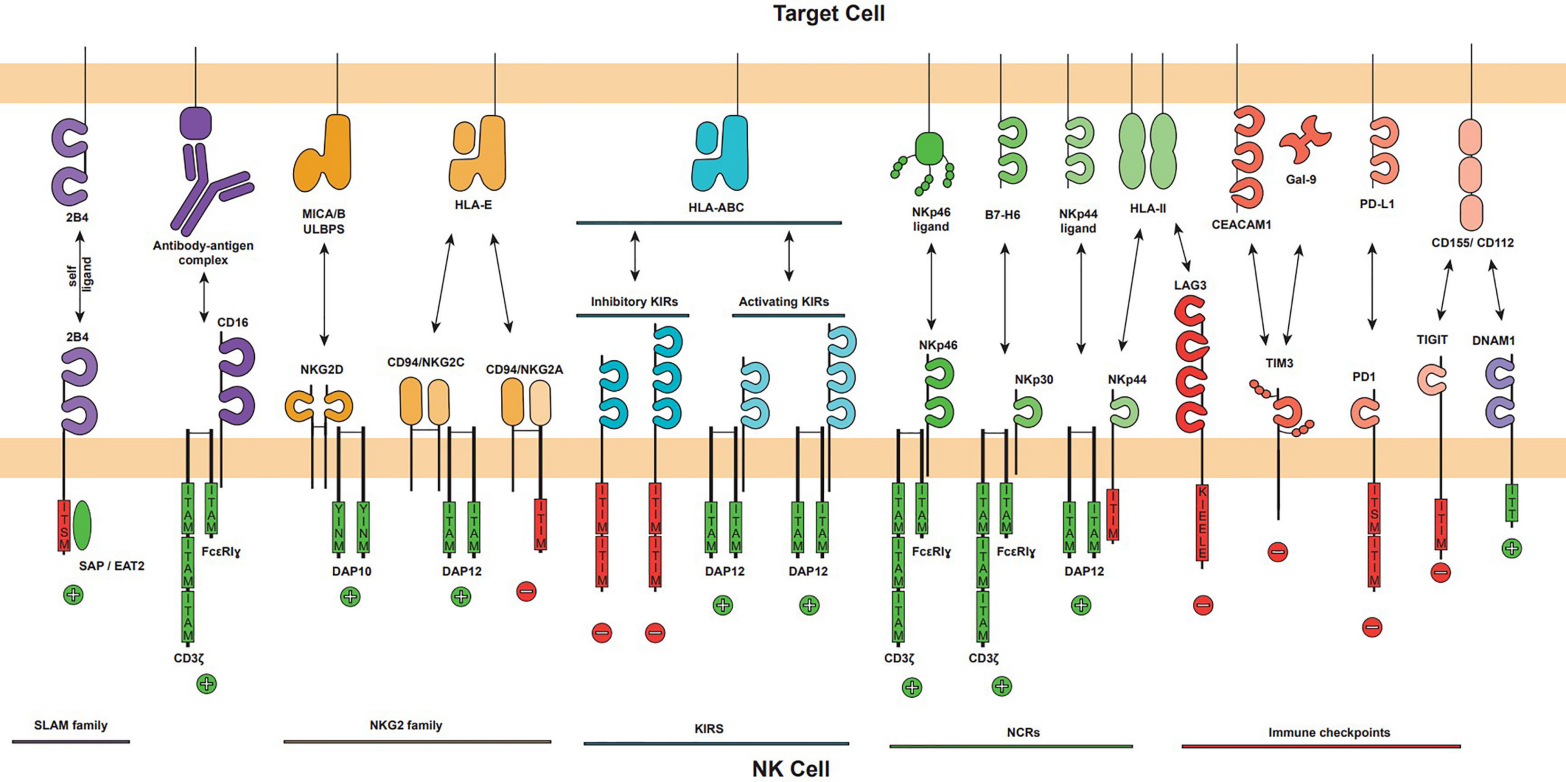
The major activating and inhibitory NK cell receptors and their ligands on target cell. The NK cell activation is mediated by the balance of activating and inhibitory signals that can trigger NK cell effector functions. NK cell receptors families are displayed (NKG2, KIR, NCR and Immune checkpoints) as well as CD16 and DNAM1 activating receptors. Each receptor is represented showing their immunoglobulin-like or lectin-like extracellular domains, its oligomerization (NKG2D homodimer, NKG2A and NKG2C heterodimer with CD94), its associated adapter (DAP10, DAP12 or CD3ζ/FcϵRIγ), and their intracellular signalling domains if applicable. Intracellular activator domains (green) ITAM and YINM promote positive activation signals, represented by a plus sign, and inhibitory domains (red) ITIM, KIEELE and ITISM trigger inhibitory signals represented by a minus sign. The major ligands of each receptor are represented.

As mentioned, NK cells present a complex germline-encoded system of inhibitory and activating receptors that help them sense microenvironmental changes due to infection or transformation, some of them related to the activation of cellular stress pathways(**Figure 1**) (14). The best characterized families for these receptors are NKG2 (NK group 2), KIR (killer Ig-like receptor), the structurally related proteins ILT/LIR (Ig-like Transcripts/leukocyte immunoglobulin-like receptor) and the natural cytotoxicity receptors (NCRs). NKG2 and KIR families are comprised of inhibitory and activating members that recognize host cell proteins, including stress ligands (NKG2D) and HLA class I molecules (NKG2A, NKG2C, ILT/LIR and KIR); while NCRs (NKp30, NK44, NKp46 and NKp80) are mostly activating receptors that recognize a broad range of pathogen and host-derived structurally diverse ligands (2). Some recent evidence indicate that some splice variants of NCRs, specially of NKp44, might work as inhibitory receptors (15, 16). The main activating and inhibitory receptors as well as their ligands are summarized in **Figure 2**. A more detailed description of these families is out of the scope of this review as it has been the topic of excellent recent reviews (17–19).

The typical structure of a NK cell receptor is an immunoglobulin-like or c-type lectin-like extracellular domain, a transmembrane domain that promotes clustering, and, some of them, mostly activation receptors, adapter proteins to signalling including CD3ζ, the Fc receptor common gamma-chain, DAP10 or DAP12. Activating receptors deliver a strong intracellular cue resulting in rapid and transient phosphorylation of the Immunoreceptor Tyrosine-based Activation Motif (ITAMs). This signal functions as ‘on and off’ switches that links the receptors to their intracellular signalling machinery working as temporal scaffolds for Src homology 2 (SH2) domains of downstream effector molecules. In the inhibitory receptors, the phosphorylated tyrosine-based inhibitory motifs (ITIMs) act as docking sites for recruiting the SHP-1 protein tyrosine phosphatase, preventing cellular activation (8).

Besides all these NK cell receptor families, other receptors have important implications in the activation of NK cell cytotoxicity. From all of them, it is worth mentioning CD16 (FCγRIIIa), the low-affinity receptor for the IgG1/3 Fc domains, with strong positive signalling in NK cells that enables the characteristic antibody-dependent cell-mediated cytotoxicity (ADCC) (20, 21). CD16 has been reported to be the most potent activating receptor of NK cell mediated target cell killing triggering degranulation (22). Other receptors like members of the SLAM family seems to be more involved in the regulation of NK-target cell adhesion than in a direct regulation of cell degranulation (23).

Thus, the balance between the intracellular signals triggered by activating and inhibitory receptors after they bind their respective ligands on target cells will dictate the activation of the intracellular cell cytotoxic machinery and the execution of the target cell. This process is triggered after the formation of a supramolecular signalling structure on the contact zone between NK and target cells known as NK cell immunological synapse (IS).

### The NK Cell Immunological Synapse Triggers the Cytotoxic Machinery

NK cell effector functions require a direct tight contact with target cells, ensuring their specific and efficient elimination. Target cell binding by receptor-ligand interaction and execution of the effector function occurs within the IS, where the stimuli triggered by NK cell receptors are integrated, resulting in a highly polarized response (24). The prototypical NK cell IS is performed through a linear sequence of required events: recognition, initiation, effector and termination. The initial stage of the immunological synapse is NK cell-target cell interaction by a receptor arbitrarily distributed over the NK cell surface, followed by establishing a strong association by adhesion molecules, mainly LFA-1 integrin (25). LFA-1 is essential due to the promotion of actin reorganization in NK cells (26). Actin polymerization and polarization are controlled, besides LFA-1, by ITAM and ITIM domains in adhesion, activating and inhibitory receptors. In the absence of prevailing inhibitory signals that can arrest progression to the effector stage, actin reorganizations mobilize the microtubule-organizing center (MTOC) to the immunological synapse while recruiting the lytic granules generating a highly polarized response (24). Then the cytotoxic granules are fused with the synaptic membrane through a process driven by SNARE family proteins like SNAPs and VAMPs (27). Synaptic cleft acts as a protective pocket, increasing lytic effector molecule concentration on target cells while protecting neighbouring cells from potential uncontrolled damage. Finally, detachment is necessary to allow cytolytic recycling of NK cells (24).

### The Main Executors of NK Cell-Mediated Killing

**Figure 3:**
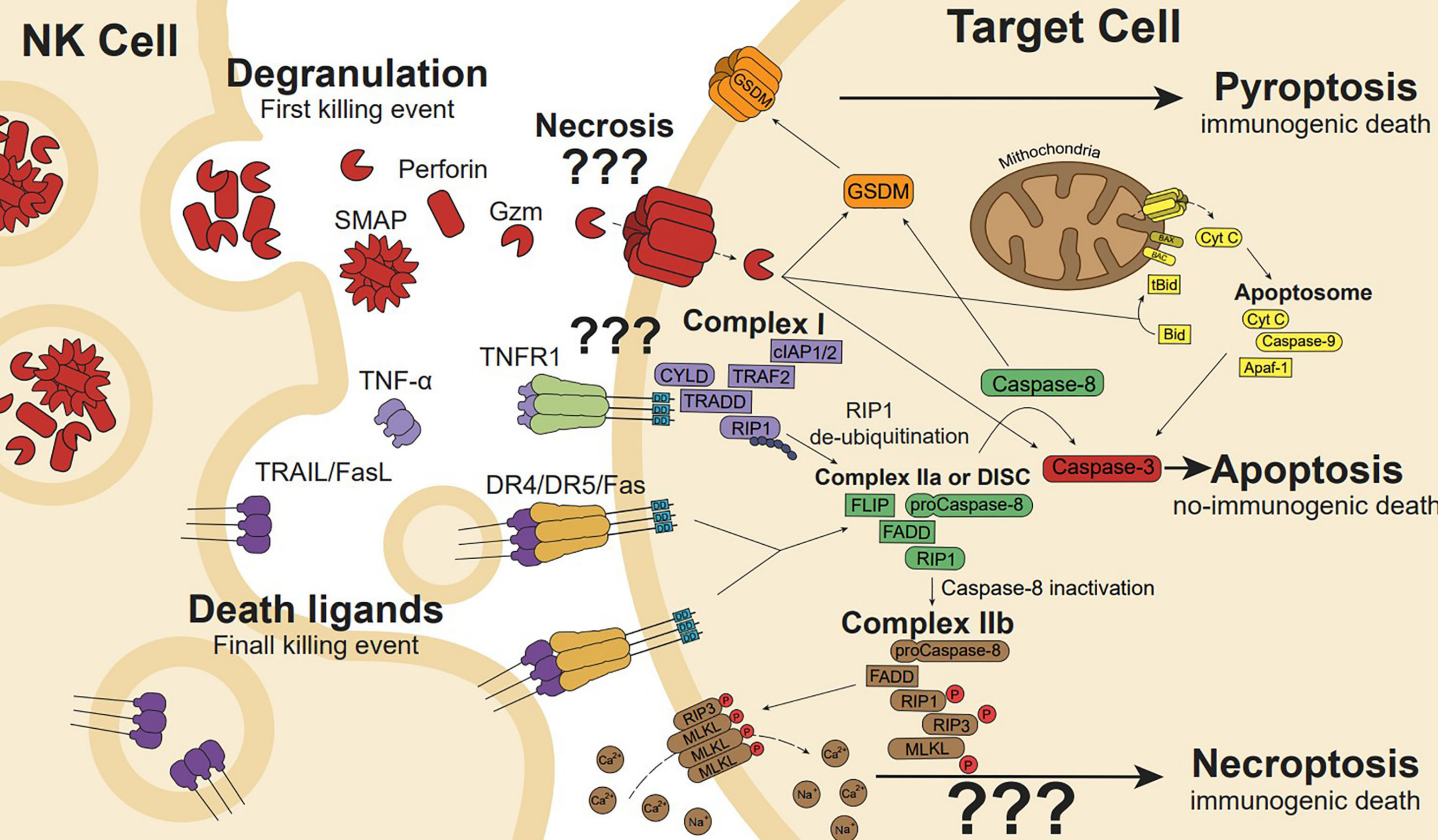
NK cell cytotoxicity is mediated by the release of cytotoxic granules and death ligands. Cytotoxic granules secretion containing perforin induces pore formation, allowing internalization of granzyme (gzm). Gzm initiates apoptosis cleaving intracellular substrates such as effectors caspase-3 and caspase-7. In addition, gzmB can cleave the BH3-only protein Bid to generate the truncated t-Bid or the anti-apoptotic protein Mcl-1 to release Bim initiating the mitochondria outer membrane permeabilization (a process known as MOMP), the release of cytochrome c and other proapoptotic factors promote the formation of the apoptosome, caspase 9 activation, and the full activation of caspase-3 and -7 to execute the apoptotic process. Gzms and Caspases can also cleave and activate gasdermins (GSDMs), linking apoptosis to pyroptosis or directly activating pyroptosis. Membrane or soluble death ligands (TNF-α, FasL and TRAIL) can induce cell death through their death receptor (TNFR1, Fas, and DR4/DR5, respectively). Ligand-receptor interaction generates receptor trimerization and its intracellular death domains clustering inducing Complex I (CYLD, TRAF2, cIAP1/2, TRADD, RIP1) formation in the case of TNFR1. RIP1 de-ubiquitination induces Complex IIa formation. However, Fas or TRAIL death ligands trimerization promotes the death-inducing signalling complex (DISC) formation, which is similar to Complex IIa in conformation and performance. In the DISC or Complex IIa, Caspase-8 auto-cleaves leading to apoptosis pathway (involving Bid and/or caspase-3), while caspase-8 inactivation induces Complex IIa transformation in IIb, which drives phosphorylated-RIP3 and MLKL ion pore-forming, resulting in ion disbalance and necroptosis cell death.

Upon activation, NK cells can kill target cells through two complex mechanisms: the release of cytotoxic granules containing perforin, granzymes and granulysin (the last only in humans), and *via* death ligands, such as Fas ligand (FasL), and TNF-related apoptosis-inducing ligand (TRAIL), (**Figure 3**) (28).

Human NK cells present specialized cytosolic granules that contain several cytotoxic proteins, including the pore-forming proteins (perforin, granulysin) and a family of serine proteases called granzymes (gzm): 5 in humans (A, B, K, M, and H) and 10 in mice (A, B, C-G, K, M, N). Gzm enzymatic activity is maximal at neutral pH, and like most proteases, gzms are synthesized as inactive zymogens (proenzymes) that must be proteolytically processed in order to become enzymatically active. All known gzms contain an inhibitory dipeptide and a N-terminal signal peptide that directs it to the endoplasmic reticulum (ER) (28, 29). The signal peptide is cleaved off, resulting in the proenzyme, which is then modified in the cis compartment of the Golgi apparatus by the N-acetylglucosamine-1-phosphodiester α-N-acetyl-glucosaminidase with a mannose-6 phosphate (M6P) moiety to provide a sorting signal that directs gzms to the secretory granules *via* the M6P-receptor (30). Once in the granule structures, M6P may be removed due to the acidic environment (pH 5.5) (31). M6P-independent pathways of sorting of gzms to granules have been described, which might involve other molecules like the proteoglycan Serglycin, although the detailed molecular mechanisms remains unclear (30, 32). Intringuingly these works found that sorting of GzmA to granules is differentially affected by Serglycin deficiency in comparison with sorting of GzmB, suggesting that trafficking and/or sorting of gzms to granules is a more complex process than previously though. Once in the granules, the cysteine proteases cathepsin C or cathepsin H remove the inhibitory dipeptide to produce the mature and enzymatically active proteases. Similar to CD8^+^Tc cells, different studies, mainly using mouse NK cells, have confirmed the role of cathepsin C or H in the generation of active gzms NK cells (33–37). Indirect evidences, using a drug that downregulates cathepsin C expression, all-trans retinoic acid, also suggest a role for cathepsin C in the activation of gzmB in human NK cells, albeit confirmation using a more specific experimental approach will be required (36). Gzms are then stored in their active form mainly associated with the proteoglycan serglycin that might act as scaffold, binding granule components to form an insoluble complex visualized as the granule core. In mouse models it has been demostrated that serglycin plays important roles in different granule-associated functions like maturation of dense-core cytotoxic granules or the trafficking and storage of perforin and gzms. In addfition, it might prevent self-damage due to the cleavage of host cell proteins (30, 32, 38). Although a direct role of serglycin in granule secretion has not been shown in human Tc/NK cells, gzmB-serglycin complexes have been identified in granules of human NK cells (38). In addition, the low pH limits gzm activity inside the granules (30, 38).

A study using mass cytometry to profile the expression of cytotoxic molecules in peripheral blood mononuclear cells found that human NK cell subsets have differential expression of cytotoxic molecules: CD56^Bright^ NK cells showed low gzmB,perforin and high gzmK expression. In contrast, CD56^Dim^ NK cells showed high gzmA, B and perforin and low gzmK expression (39). Other studies by flow cytometry indicated that CD56^Dim^ cells have at least ten times more perforin and gzmA than CD56^Bright^ ones (40, 41). However, these results might depend on the pathology and the tissue where they were gathered since it was recently shown that CD56^Bright^ cells in the synovium of osteoarthritis patients express high levels of gzmA, similarly to those found in synovial CD56^Dim^ NK cells. In contrast, the level of gzmB in synovial CD56^Bright^ cells was significantly lower than in synovial CD56^Dim^ NK cells (42). The high expression of gzmA in the non-cytotoxic CD56^Bright^ subset supports recent findings indicating that gzmA is involved in processes unrelated to cell-mediated cytotoxicity, like the regulation of the inflammatory responses and extracellular matrix remodelling (8, 13, 43–48).

In contrast to gzms, that are expressed by different immune cell populations, including cytotoxic and non-cytotoxic cells (44), perforin is uniquely expressed in cells with cytotoxic potential such a NK cells. It is a glycoprotein that, in the presence of calcium, has the ability to insert into lipid bilayer membranes, polymerize and form structural and functional pores allowing gzms delivery into the target cell. Perforin deficient mice are more sensitive to tumour development, including NK-cell sensitive tumours (10, 12, 44). In contrast the role of NK cell-associated gzms in the control of tumour development is still not clear. Some studies have found that gzmA/B KO mice are more susceptible to NK-sensitive tumours than wt mice, while others have found no difference (49, 50).

As indicated above other molecules within cytotoxic granules, like serglycin, regulate the cytotoxic function of perforin and gzms. Recently, it has been described in human NK and CD8+Tc cells that the release of perforin and gzmB is performed in approximately equal amounts of soluble and stable complexes, called supramolecular attack particles (SMAPs) which were composed of a core shell structure and were assembled in the dense secretory granules before release (51, 52). The SMAPs, that were found in both human CD8+Tc and NK cells, consist of a core of gzms, perforin and serglycin proteoglycans, and galectin-1 surrounded by a glycoprotein shell that includes Thrombospondin 1 (TSP-1), a Ca^2+^-binding glycoprotein (52). It is notable to point out that these studies have been performed in human cells. When the content of lytic granules is released into the IS, the subsequent uptake of gzms by the target cell through pores generated by perforin initiates the death program that will eventually lead to cell elimination. The gzm and perforin complexing within SMAPs may offer a mechanism to increase localized perforin concentrations in target cell membranes or prevent toxic proteins from leaking out of the synaptic cleft. Although alternative models have been proposed, such as the internalization of the gzm–Prf–Srgn complexes in endosomes (53, 54).

More recent experimental evidence indicate that perforin-mediated pore formation on the target plasma cell membrane is the key event for the intracellular delivery of gzms (55–57). Although perforin-independent cytotoxic functions of gzms have been described, their relevance during NK cell-mediated target cell killing is not clear and will not be discussed here (44).

### Death Ligands

In addition to NK cell-mediated killing by cytotoxic granules, activated NK cells can induce cell death in target cells through activating the death receptor pathway. Here a protein, known as death ligand, produced by the effector cell binds to the respective receptor expressed on the target cell membrane triggering target cell death. NK cells express different death ligands with potential cytotoxic activity like TNFα, Fas ligand (FasL), and/or TRAIL. However, from all of them, only FasL and TRAIL have been shown to act as direct cytotoxic molecules during NK-cell mediated cell killing in humans and mice. The use of one or more ligands by NK cells seems to be influenced by the susceptibility of the target cell and/or the stage of NK cell maturation and/or activation (58, 59).

These ligands belong to the TNF family and are naturally expressed by immune cells, including NK cells, granulocytes, monocytes, T cells, B cells and dendritic cells, among others (60). They are transmembrane proteins that can be proteolytically cleaved and released in a soluble form or forming part of microvesicles (61–67). In contrast to soluble FasL that does not present bioactivity, both soluble TRAIL and TNF-α mediate different biological activities including killing of cancer cells (68). Death receptors are type I transmembrane proteins with extracellular domains rich in cysteine and a 80-amino acid conserved sequence in the cytoplasmic domain called death domain (DD). When the ligands are bound to their receptors, DD induces receptor trimerization and recruitment of Fas-associated death domain (FADD)/caspase-8 complex, which triggers cell death (**Figure 3**). In addition, to activate different cell death modalities, depending on the composition of the signalling complexes formed after ligand-receptor interaction, some of these receptors can activate the NF-kB pathway involved in pro-survival signalling, proliferation and/or cytokine production, depending on the composition of the signalling complexes formed after ligand-receptor interaction (69–72).

TNFα is a type II transmembrane protein that is cleaved and released as a soluble form after processing by TNF-α-converting enzyme (TACE), that mediates its biological activity by binding to TNF receptor 1 (TNF-R1 or DR1) and TNF receptor 2 (TNF-R2). TNF-R1 is classified as a death receptor since it possesses a DD, so it can trigger cell death under certain conditions, whereas TNF-R2 lacks a DD and belongs to the non-death receptor group of the TNF receptor superfamily (TNFRSF).

FasL (CD95L), a type II transmembrane protein, is expressed in NK cells and interacts with DR2 (Fas or CD95) and DcR3 (73, 74). DcR3 inhibits FasL/Fas activity, thus acting as a fake receptor (75). However, when FasL binds to Fas, it starts the clustering of the receptor leading to cell death. TRAIL, as FasL, is a transmembrane protein type II and modulates the immune response (76). TRAIL can bind to three decoy receptors: DcR1(TRAIL-R3), DcR2(TRAIL-R4), OPG, and two DR: DR4 and DR5. TRAIL is known to induce apoptosis in transformed cells while sparing the non-transformed ones (77, 78).

In addition to their role in regulating peripheral tolerance and immune cell homeostasis, FasL and TRAIL have been shown to contribute to NK-cell mediated cell killing and tumour immunosurveillance in mice and humans (3, 58, 59, 73, 79, 80).

As indicated above, the use of TNFα by NK cells as a molecule with direct cytotoxic capacity against cancer cells is not clear. However, it has been shown to modify the cancer cell susceptibility to NK cells by modulating the expression of molecules involved in target cell recognition and IS formation like members of ICAM family (81, 82).

### NK Cell Effector Function Dynamics

As mentioned above, NK cell cytotoxicity is mediated by the directed release of preformed cytotoxic granules and the expression of death ligands that activate their respective death receptors on the surface of the target cells. Although it is clear that NK cells can use both pathways to kill tumour cells *in vitro*, the relative contribution of each mechanism to the elimination of target cells and during tumour immunosurveillance *in vivo* is still not clear, and likely dependent on the sensitivity of tumour cells to each mechanism (83). Former studies in perforin deficient mice showed that perforin is the main executor of NK-cell mediated cell killing and cancer immunosurveillance (10, 49, 50, 84–86). However, later on, it was shown that TRAIL is required for the control of tumour metastasis (87) and, more specifically, it contributed to NK cell-mediated control of cancer metastasis (79, 88). Regarding FasL, indirect evidence using tumour cells expressing inhibitors of the death receptor pathway like CrmA (a cowpox protein) or FLIP, suggested that FasL was also involved in NK cell-mediated tumour immunosurveillance (89, 90). However, in this case, it could not be differentiated between FasL and TRAIL since CrmA and FLIP overexpression blocks both pathways.

More recently, it has been proposed that these two distinct cytotoxic mechanisms were shown to act on different time scales, with rapid granule-mediated cell death and slower death ligand-induced cell death. Indeed, it has been shown that the contribution of perforin and death ligands to NK cell-mediated killing might be related to the ability of a single NK cell to kill more than one target cell, a process known as serial killing (91–93). Indeed, Deguine et al., using intravital microscopy demostrated that NK cells form transient contacts with tumor cells, compared with the more stable contact of Tc cells, allowing NK cells to establish multiple contacts over short periods favoring the serial killing capacity that has been described *in vitro (* 94). A similar result was obtained by Halle et al. using a model based on Tc cell-dependent cytotoxicity, confirming that the serial killig capacity of NK cells is more pronounced than that of Tc cells (95).

After target cell death, the locally attached NK cell can disengage and subsequently mediate additional killing events of neighbouring target cells (91, 93). The two mechanisms (cytotoxic granules and death receptor-mediated killing pathways) are coordinated and regulated during the serial killing activity of NK cells. It was shown that NK cells contained an average of 200 cytotoxic granules and released about 10% of their total granules in a single killing event. They require about 1% of cytotoxic granules to kill a target cell, suggesting that NK cell cytotoxic granules are highly efficient and that NK cell do not release their entire lytic granule reserve onto a single target cell allowing NK cells to perform serial killing of multiple target cells. However, not all NK cells have the serial killing capacity, being about the 10% of the entire NK cell population responsible for 30% of target cell death (93, 96). Although death receptor signalling is possibly initiated simultaneously with granzyme activity, the fast and efficient granule-mediated cell death is likely dominant in the initial stages over the slower death receptor pathway (97). Only after increasing concentrations of surface death ligands accumulate on the NK cell membrane, it becomes more prominent so that the final kill events are dominated by death receptor stimulation. In other words, for their first killing events, NK cells almost exclusively use the granule-mediated pathway, resulting in a swift and efficient killing of target cells. After losing some of granule content and increasing surface Death Ligands, NK cells switch from granule to death ligand-mediated cytotoxicity. The complementary action of death ligands and cytotoxic granules, co-expressed at varying levels among individual NK cells, could facilitate the lytic action of even poor perforin/gzm-expressing NK cells. They can synergize, be additive, or act complementarily (97). Thus, it is possible that under specific conditions where perforin cannot act like genetic deficiency (perforin KO mice or human type 2 FHL) or in cancer cells with perforin resistance, death ligands would contribute to NK cell-mediated cancer control. In addition, the expression of intracellular inhibitors that might regulate the susceptibility of cancer cells to granule exocytosis and/or death receptor pathways will dictate whether the contribution of each pathway to NK-cell mediated tumour cell killing and cancer progression.

## Second Act: The Execution Phase or how Target Cell Undergoes NK Cell-Mediated Programmed Cell Death

Most studies analysing target cell death pathways activated by perforin/granzymes or death ligands have been performed using purified recombinant or native proteins and, in some cases validated mainly using CD8^+^Tc cells. These seminal studies have been key to properly understand the role of cell-mediated cytotoxicity and programmed cell death pathways in pathogen and tumour control. However, when extrapolating these findings to NK cell-mediated cytotoxicity, it should be taken into account that the precise expression of effector molecules and how they are activated during NK cell-mediated elimination of target cells might influence the cell death pathways activated in the target cells. In addition, as indicated above, the cytotoxic mechanisms that NK cells use to trigger tumour cell death cannot be viewed as independent events. For example, it is known that the different cell death pathways that are activated by these mechanisms such as apoptosis, necroptosis or pyroptosis interact with each other. On the other hand, apoptosis has been described as non-immunogenic canonical cell death due to the formation of apoptotic bodies that enclose intracellular content, while pyroptosis and necroptosis have been described as immunogenic programmed cell deaths, resulting in the spillage of cellular content, like necrosis that is an unregulated form of cell death mainly caused by cell injury or trauma. In addition, granule exocytosis might regulate the susceptibility to death receptors and vice versa.

Next, we will discuss the main cell death pathways that have been described to be activated by granule exocytosis and death ligands although since the information regarding NK cells is scarce, on some occasions, we will refer to cell death mechanisms found using pure proteins or CD8^+^Tc cells.

### Programmed Cell Death Pathways Activated by Granule Exocytosis

As discussed above, once granule content is released in the NK IS, perforin facilitates the intracellular delivery of gzms into the target cell. Perforin-mediated self-damage on NK cell membrane would be prevented by different mechanisms like CD107a, a lysosomal/granule protein that is translocated to the NK cell membrane during degranulation. However recently this mechanisms has been disputed by Rudd-Schmidt et al, 2019 describing two protective properties of the plasma membrane of cytotoxic lymphocytes within the synapse, an increased plasma membrane lipid order, thus reducing perforin binding, and the exposure of negative charge on the membrane surface *via* phosphatidylserine, thus inactivating residual perforin within the immune synapse (98–100). Once delivered into the target cell, gzms would cleave intracellular substrates leading to the target cell death. Among all gzms, gzmB is the one with a highest cytotoxic potential, while the ability of other gzms to kill target cells is still controversial and out of the scope of this review (8, 10, 101, 102). Although extracellular gzmB has also been involved in perforin-independent cell death *via* receptor activation in neurons or after extracellular matrix degradation in fibroblasts and smooth muscle cells (103–105), we will not focus here in this pathway as its relevance during NK cell mediated cytotoxicity has not been explored.

Both, human and mouse GzmB initiates apoptosis cleaving several intracellular substrates such as effectors caspase-3 and caspase-7 (106–108).In addition, human and mouse gzmB can cleave the BH3-only protein Bid in human and mouse target cells, respectively, to generate a truncated t-Bid form (109–113) and the Bcl-2 protein, Mcl-1, which is degraded releasing the pro-apoptotic protein Bim (114, 115). Here it should be noted that studies using purified proteins have shown that the substrate affinity of mouse and human gzms are different, presenting mouse gzmB a higher affinity for mouse caspase-3 than for mouse Bid, while the opposite was shown for human gzmB (116, 117). However the relevance of these findings during cell death induced by pure gzmB or gzmB of NK/Tc cells on intact target cells is not well understood and seems to be more complex than suggested by the substrate affinity studies. This assumption is supported by functional studies using mouse target cells with deficiency in caspase-3 or Bid pathways showing that the absence of caspase-3 or Bid influences the molecular mechanism of apoptotic cell death induced by mouse gzmB (109, 110) suggesting that both pathways contribute to gzmB-mediated cell death in both human and mice. Both pathways initiate the mitochondria outer membrane permeabilization (a process known as MOMP), the release of cytochrome c and other proapoptotic factors like SMAC/Diablo, promoting the formation of the apoptosome and the full activation of caspase-3 and -7 to execute the apoptotic process (8, 10–12, 43). Alternatively, human and mouse gzmB can also induce DNA fragmentation through its ability to cleave the cytoplasmic nuclease inhibitor, ICAD, releasing the Caspase-activated DNase, CAD, that promotes DNA degradation (118, 119). In addition, it cleaves other proteins involved in the maintenance of nuclear integrity (Lamin B), DNA repair (DNA-PK_cs_), poly(ADPribose) polymerase (PARP), microtubule dynamics (*α*-tubulin), and host autoantigens (NUMA, U1-70kD, Mi-2) (8, 10, 102, 107, 108, 120, 121). Here it should be noted that some of these studies were performed using either mouse or human gzmB, and confirmation in both species might be required to understand the biological relevance of these findings.

Albeit gzmB activates apoptosis by direct or indirect mitochondrial-mediated caspase activation, it has been shown that these pathways are dispensable for the elimination of tumour cells mediated by gzmB of Tc and NK cells, both in mouse and human models, *in vivo* and *in vitro (* 109, 110, 122, 123). Whether NK cell mediated-tumour cell death in the absence of caspases and the mitochondrial pathway is executed by some of the gzmB substrates indicated above or by the activation of other cell death pathways is still being explored. For example some of the gzmB substrates indicated above (DNA-PKs, NUMA, lamins or ICAD) have been validated using LAK cells, which are NK cells generated *in vitro* using a high concentration of IL2 (108, 119, 121).

Alternatively, recent studies have shown that human and mouse gzmB released by NK cells could also directly cleave and activate gasdermin E (GSDME) in a caspase-independent manner, promoting pyroptotic cell death (9, 124). Pyroptosis is an inflammatory cell death modality regulated by the GSDM family. GSDMs are activated by cleavage, forming a transmembrane pore that contributes to IL1 family cytokine release and, in addition, disturbs intracellular ion homeostasis, resulting in cell death (125). There are different members on the GSDM family, all of which are activated by caspase-mediated cleavage. Traditionally GSDM activation was associated to inflammatory caspases like caspases-1, -4 or -5. More recently, it has been shown that other caspases, like caspase-3 or -8, can activate different GSDMs in Gsdm-expressing tumour cells. Furthermore, it has been recently shown that during GzmB-mediated cell death, caspase-3 is activated and mediates Gsdm cleavage and pyroptosis linking the gzm pathway to cell pyroptosis (124). The main role of pyroptosis seems to be inducing strong inflammatory responses that contribute to host defense against pathogen infection.

Regarding gzmA, the other major granule protease, its cytotoxic potential is reduced in comparison with gzmB, and at present, the relevance of cell death induced by gzmA is still in debate (8, 10, 46, 102, 126, 127). Recently it was shown that human gzmA released from NK cleaved and activated GSDMB, triggering pyroptosis in a caspase-independent manner (128).

### Programmed Cell Death Pathways Activated by Death Receptors

Activation of the death receptor pathway by death ligands can also kill target cells through different cell death modalities, including apoptosis, necroptosis or pyroptosis. After a death receptor is engaged by its respective ligand, the adaptor protein FADD is recruited to the cytosolic DD of the receptor *via* homotypic interactions. Then the death effector domain (DED) present in FADD recruits procaspase-8, FLIP, and RIP1 to form a death-inducing-signalling complex, known as DISC in the case of TRAIL and FasL (70, 129) (**Figure 3**). Within the DISC, molecules of procaspase-8 in close proximity undergo autocatalytic cleavage to stabilize caspase-8 in its catalytically active conformation that will activate downstream cytosolic effector caspases, including caspase-3 and caspase-7. Under some circumstances, death receptor stimulation is insufficient to induce apoptosis *via* direct cleavage of caspase-3 and, then, caspase-8 mediated activation of Bid and the mitochondrial apoptotic pathway is required as described for gzmB (70, 71, 130).

Similar to FasL and TRAIL, the engagement of TNF*α* to TNFR1 generates the formation of a large complex known, in this case, as Complex I (74, 82) (**Figure 3**). It includes cIAP1, cIAP2, CYLD, RIP1, and TRAF2. While cIAPs induce RIP1 polyubiquitination inhibiting Complex IIa formation, CYLD promotes its deubiquitination, boosting Complex IIa generation and RIP1-mediated apoptosis. Furthermore, within Complex IIa, activated caspase-8 cleaves and inactivates RIP1, RIP3, and CYLD. Cleaved RIP1 and RIP3 lose their transphosphorylation and downstream substrate phosphorylation capabilities (74). However, when the cleavage of RIP1 and RIP3 is prevented by caspase-8 inhibitors or by the genetic deletion of caspase-8 or FADD, Complex IIb is formed (**Figure 3**). In Complex IIb, MLKL is phosphorylated by RIP3, oligomerising and translocating to the cell membrane, where it binds to phosphatidylinositides inducing cell membrane disruption and necroptotic cell death (69, 71, 129, 131).

Although all death ligands present differences in their signalling cascades, they can induce a multi-protein complex formation involving procaspase-8, FLIP, FADD, and RIP1. In this way, both TRAIL and FasL can also induce necroptosis in specific conditions, such as when caspase-8 is inhibited (71). The formation of each complex is regulated by a complex network of protein interactions from host cells and pathogens, including FLIPs and IAPs, that modulate cell outcome (survival or death) and the modality of cell death (69).

Similar to granule exocytosis, the relevance of the cell death pathways activated by death ligands during NK cell-mediated cytotoxicity is still not clear. It might also be influenced by the profile of death ligand expression in NK cells and the strength of the signals received by NK cells during target cell recognition. Curiously, it was recently shown that NK cells were able to activate necroptosis in some target cells. However, surprisingly, this process was not mediated by death receptors but by the gzmB pathway. This result was supported by previous findings showing that during NK cell attack, apoptosis, necrosis and mixed forms of cell death could be detected in target cells (132). In this line, Prager et al. showed that human NK cells used both gzmB and FasL to activate apoptotic- and/or necrotic like morphology in target cells (97). Further experiments will be required to determine whether this necrotic-like morphology is necroptosis or, for example, perforin-mediated lysis. Here it should be clarified that most of these results were mainly observed using gzmB inhibitors with no clear specificity, and, thus, further experimental work will be required to validate them using more specific approaches like CRISPR/Cas9-mediated knock-down. More importantly, despite NK cells might express high amounts of TNFα, it is still not known whether NK cells can activate classical necroptosis in cancer cells by using TNFα.

Thus, albeit it has been proposed that specific mutations in some of these cell death pathways might contribute to tumour cell resistance to NK-cell mediated activation of cell death (133), confirmation using NK cells is mandatory to find out the relevance of these mutations during NK cell mediated cancer immunosurveillance.

## Death is not the end: NK Cell-Mediated Cell Death Boosts Adaptive Immunity

The primary role of NK cells, as indicated above, is to eliminate infected and transformed cells by inducing target cell death. Albeit apoptosis has been considered the paradigm of programmed cell death, more recently it has been confirmed that NK cells are also able to induce other forms of cell death like necroptosis and pyroptosis. These more inflammatory ways of cell death can lead to an increased release of both DAMPs and tumour antigens promoting inflammation and potentially the activation of adaptive immune responses against endogenous cancer antigens, a process known as immunological cell death (ICD) (122, 134, 135).

Thus, cell death activation in tumour cells by NK cells could be an additional mechanism that links the innate to the adaptive immune responses in cancer immunity (72).This mechanism would complete the cancer-immunity cycle (136, 137) linking an initial elimination of some tumour cells by NK cells to the activation of T cell responses that would complete tumour cell elimination (138). This hypothesis has been recently confirmed experimentally *in vivo* in mouse models showing that both NK and Tc cells induce ICD in primary tumours promoting the activation of new Tc cell responses against antigens released by dying tumour cells, which prevents the growth of secondary tumours (139, 140).

These findings increase our understanding about the role of NK cells in shaping tumour adaptive immune response and support previous results indicating that NK cells drive inflammation and immune cell infiltration, including conventional type 1 Dendritic Cells (cDC1), increasing tumour neoantigen presentation and CD8^+^ T cell immunity (141–144).

The specific role of apoptosis, necroptosis, and pyroptosis in NK cell-mediated ICD is still unknown. However, the results obtained using Tc cells *in vivo* indicate that the presence of active caspase-3 is required for ICD, at least when induced by Tc cells. Tc-cell mediated killing of tumour cells expressing a dominant negative mutant of caspase-3 were eliminated as efficiently as wild type tumour cells, but ICD determinants were reduced in mutant caspase-3 cells, and the protection against a secondary tumour challenge was lost (139). This result indicates that caspase-3 dependent apoptosis in the context of effector cell attack is immunogenic or, alternatively, ICD is activated due to secondary pyroptosis as a consequence of caspase-3 mediated GSDME activation as recently shown (9, 124). Further experimental analysis will be required to confirm these hypotheses. Here it should be considered that exacerbated effector cell-mediated responses might also be detrimental and promote cytokine release syndrome (CRS) as recently shown when using highly activated CAR-T cells, expressing high amounts of gzmB, which activates GSDME dependent pyroptosis, contributing to CAR-T toxicity. Interestingly, unlike CAR-T cells, CAR-NK cells do not promote CRS, pointing to a more controlled immune response during target cell elimination (145).

At present, the role of necroptosis in ICD induced by NK cells has not been explored.

As indicated above it is not clear at what extent mutations in different cell death pathways contribute to evasion of NK cell responses. This scenario becomes even more complex when considering that these mutations might not affect killing of primary tumour cells, but they might impact the immunogenic characteristics of cell death, reducing the activation of adaptive T cell responses, which in turn would reduce the elimination of primary tumour cells and the generation of immune memory against recurrent cancer cells.

## Conclusion

NK cells are key mediators of cellular cytotoxicity, which is an important effector mechanism of the immune system (28, 146). The ability to directly kill other cells is critical for removing infected or transformed cells and is, therefore, a central tool in the immune system’s fight against viral infections and cancer. It is well established that the different cytotoxic effector mechanisms of NK cells, including granule exocytosis and death ligands, are essential for the elimination of tumors (147, 148). Here the pleiotropic ability of these mechanisms to activate different cell death programs in the target cells is essential for the elimination of offending cells, especially to overcome potential mutations in the cell death machinery that might compromise target cell elimination. Moreover, the most recent evidences indicate that NK cell mediated cytotoxicity is not only involved in the control of initial tumour development, but, in addition, regulates the activation of adaptive T cell responses by inducing ICD. ICD provides inflammatory signals and antigens to activate and expand new anti-tumoral T cells, enhancing the efficacy of tumour elimination and, potentially, preventing cancer metastasis and recurrence. Thus, on a conceptual basis, NK cell-mediated cell death might be considered the initial signal that triggers the cancer-immunity cycle. Here future efforts should be directed to understand the role of mutations in cancer cell death machinery on ICD that might impact on cancer metastasis and recurrence.

The information about the role of the different cell death mechanisms activated by NK cells on the control of tumor development and the efficacy of the different types of immunotherapy is still limited. However, all the new findings discussed here point to a fundamental role for NK cell-mediated cytotoxicity in the efficacy of these new treatments, including checkpoint inhibitors and adoptive cell transfer (i.e. allogeneic NK cells and CAR-NK). A better understanding of the role of apoptosis, necroptosis, and pyroptosis in NK cell-induced death will allow us to design better treatments to eliminate tumors and prevent recurrences effectively and safely, reducing the potential adverse effects of immunotherapy like Cytokine Release Syndrome.

## Author Contributions

AR-L and CP elaborated the manuscript and the figures, and curated the bibliography. LS, SH, AC, CO, AA-T, MG-T, IU-M, MA, and EG participated during the draft elaboration and review of the manuscript. JP provided the main view, revised the elaboration process and finalized the manuscript. All authors contributed to the article and approved the submitted version.

## Funding

This research was supported by CIBER -Consorcio Centro de Investigación Biomédica en Red- (CB 2021), Instituto de Salud Carlos III, Ministerio de Ciencia e Innovación and Unión Europea – NextGenerationEU, FEDER (Fondo Europeo de Desarrollo Regional, Gobierno de Aragón (Group B29_20R), Ministerio de Ciencia, Innovación e Universidades (MCNU), Agencia Estatal de Investigación (SAF2017-83120-C2-1-R; PID2020-113963RB-I00), ASPANOA, Donación Javier Saura Carceller, and Carrera de la Mujer de Monzón. JP was supported by Fundacion Aragon I+D (ARAID). MA was granted by a Juan de la Cierva contract of Spanish Ministry of Economy and Competitiveness (IJC2019-039192-I). LS was granted by a Juan de la Cierva contract of Spanish Ministry of Economy and Competitiveness (FJC2020-046181-I). IU-M and SH are supported by a PhD fellowship from Aragon Government, CP by a PhD fellowship from Fundación Científica de la Asociación Española Contra el Cáncer (FC AECC), MG-T by a PhD fellowship (FPI) from the Ministry of Science, Innovation and Universities. CO was granted by AEI -Agencia Estatal de Investigación- (PTA2020-018510-I / AEI / 10.13039/501100011033).

## Conflict of Interest

The authors declare that the research was conducted in the absence of any commercial or financial relationships that could be construed as a potential conflict of interest.

## Publisher’s Note

All claims expressed in this article are solely those of the authors and do not necessarily represent those of their affiliated organizations, or those of the publisher, the editors and the reviewers. Any product that may be evaluated in this article, or claim that may be made by its manufacturer, is not guaranteed or endorsed by the publisher.
